# Supplementary material for: Broadly conserved FlgV controls flagellar assembly and Borrelia burgdorferi dissemination in mice
Source: Nat Commun. 2024 Nov 29;15:10417. doi: 10.1038/s41467-024-54806-w (PMC11607428; doi:10.1038/s41467-024-54806-w)
Supplement: Supplementary file 1 — Supplementary Information [file 41467_2024_54806_MOESM1_ESM.pdf]

SUPPLEMENTARY INFORMATION

Broadly conserved FlgV controls flagellar assembly and *Borrelia burgdorferi* dissemination in mice

Maxime Zamba-Campero[1,†], Daniel Soliman[1,†], Huaxin Yu[2,3], Amanda G. Lasseter[4], Yuen-Yan Chang[1], Julia L. Silberman[1], Jun Liu[2,3], L. Aravind[5], Mollie W. Jewett[4], Gisela Storz[1], and Philip P. Adams[1,4,6,7*]

[1]Division of Molecular and Cellular Biology, *Eunice Kennedy Shriver* National Institute of Child Health and Human Development, National Institutes of Health, Bethesda, MD 20892, USA.

[2]Department of Microbial Pathogenesis, Yale School of Medicine, New Haven, CT 06536, USA.

[3]Microbial Sciences Institute, Yale University, West Haven, CT 06516, USA.

[4]Division of Immunity and Pathogenesis, Burnett School of Biomedical Sciences, University of Central Florida College of Medicine, Orlando, FL, 32827, USA.

[5]National Center for Biotechnology Information, National Library of Medicine, National Institutes of Health, Bethesda, MD 20894, USA.

[6]Postdoctoral Research Associate Program, National Institute of General Medical Sciences, National Institutes of Health, Bethesda, MD 20892, USA.

[7]Independent Research Scholar Program, Intramural Research Program, National Institutes of Health, Bethesda, MD 20892, USA.

[†]equal contribution

*correspondence: philip.adams@nih.gov

SUPPLEMENTARY FIGURES

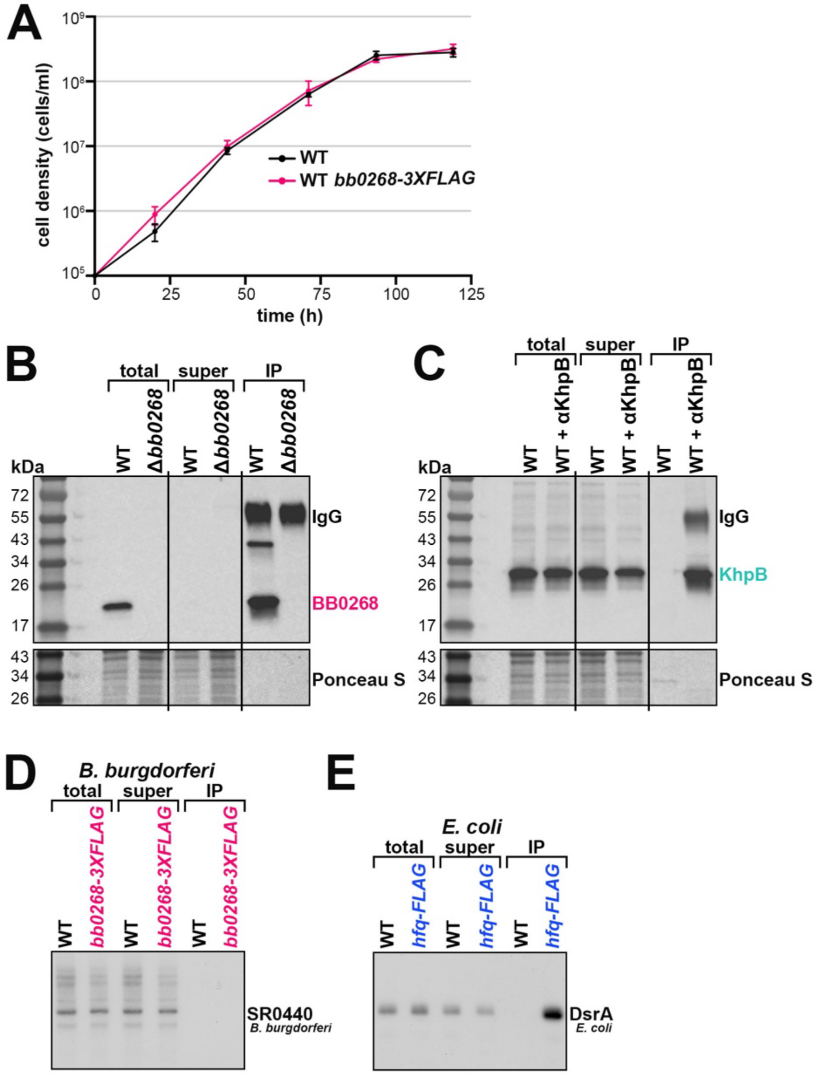

Fig. S1. BB0268-3XFLAG and native BB0268 does not bind RNA. (**A**) Growth curve of *bb028-3XFLAG* (PA007) and WT (PA003) *B. burgdorferi*. Cell growth was monitored by dark field microscopy enumeration at the indicated time points, after dilution of the starter culture to 1x10^5 cells/ml. Each data point (circles) represents the mean of three biological replicates with the standard deviation. (**B**) Immunoblot analysis of immunoprecipitated WT *B. burgdorferi* (PA001) or Δ*bb0268 B. burgdorferi* (PA251) samples. *B. burgdorferi* cells were grown to ~6.3x10^7 cells/ml and then exposed to UV to crosslink any RNAs associated with the proteins. After cell lysis, the tagged proteins were immunoprecipitated (IP) and RNA was isolated from

the IP samples. Equal volumes of cell lysis (total), supernatant of the α-BB0268-beads during washing, and elution from the α-BB0268-beads (IP) were separated on a Tris-Glycine gel, transferred to a membrane, stained with Ponceau S, and probed using α-BB0268 antibodies. Size markers are indicated. The IgG label denotes the heavy chain of the BB0268 antibody. (**C**) Immunoblot analysis of immunoprecipitated WT *B. burgdorferi* (PA001) using KhpB antibodies. *B. burgdorferi* cells were grown to ~$8.1x10^7$ cells/ml and co-IP performed, as in panel B. The immunoblot was probed using α-KhpB antibodies. Size markers are indicated. The IgG label denotes the heavy chain of the KhpB antibody. **(D)** Northern analysis of BB0268-3XFLAG RNA samples. One microliter of each *B. burgdorferi* sample from Fig. 1A was separated on an acrylamide gel, transferred to a membrane, and probed for *B. burgdorferi* DsrA. (**E**) Northern analysis of *E. coli* Hfq-FLAG RNA samples from Fig. 1A. Samples were analyzed as in panel D and probed for *E. coli* DsrA.

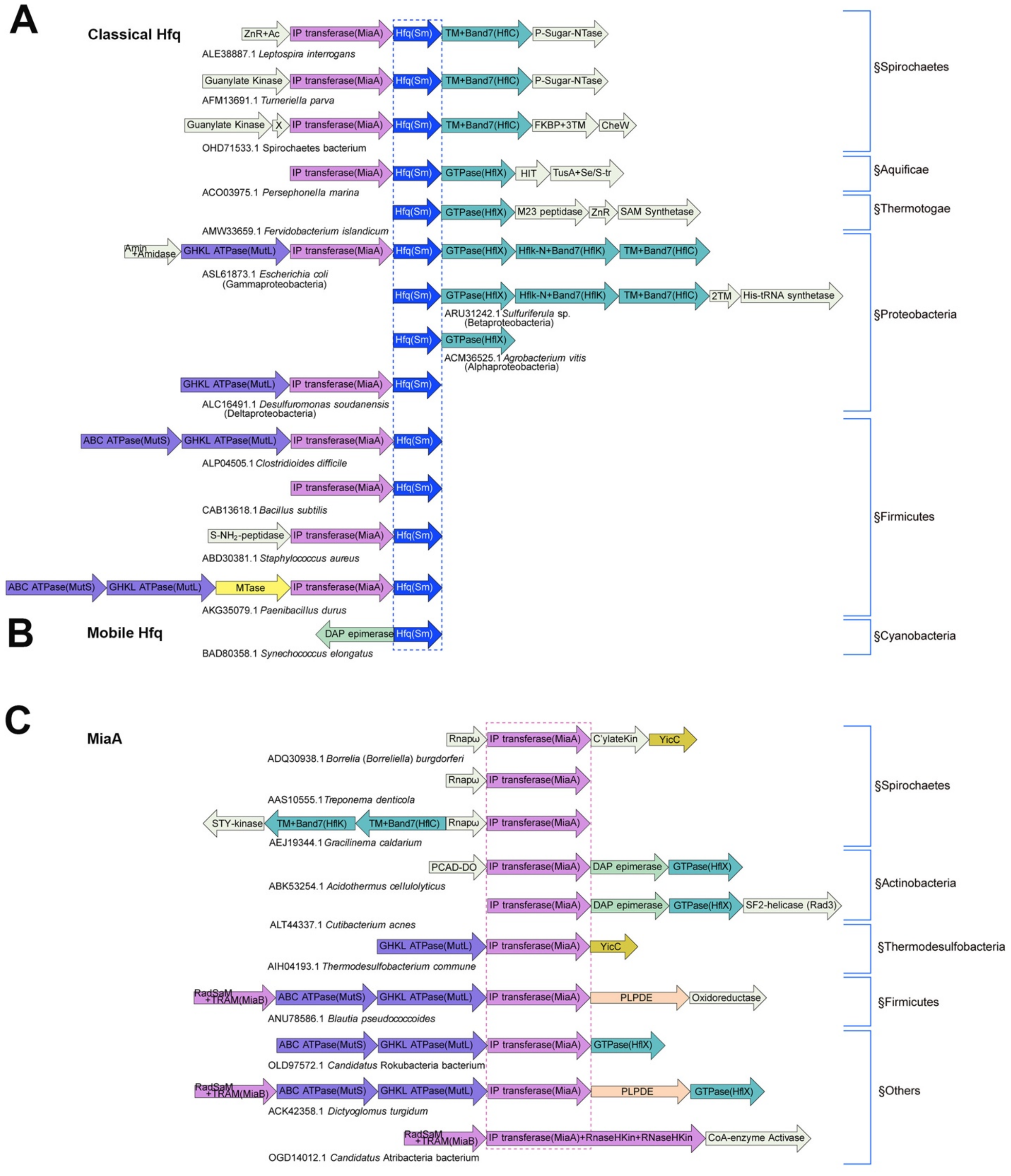

Fig. S2. The conserved *hfq* and *miaA* gene neighborhoods. Gene neighborhoods from representative species from across the bacterial tree of life for: (**A**) The classical *hfq*, the homohexameric RNA binding protein of the Sm domain superfamily, clade; (**B**) The mobile *hfq* clade; (**C**) The *miaA*, the tRNA adenine isopentenyl transferase, clade. For panels A–C: the

genes are shown as box arrows (only drawn to approximate size) and labeled with the domain architectures of the encoded proteins. Each operon is labeled, below the box arrows, based on the GenBank accession of the anchor gene and the species name. All distinct core genes of the neighborhood are colored differently, and the anchor genes are marked with a dashed box. The genes specific to particular operons but not otherwise widely conserved are colored off-white.

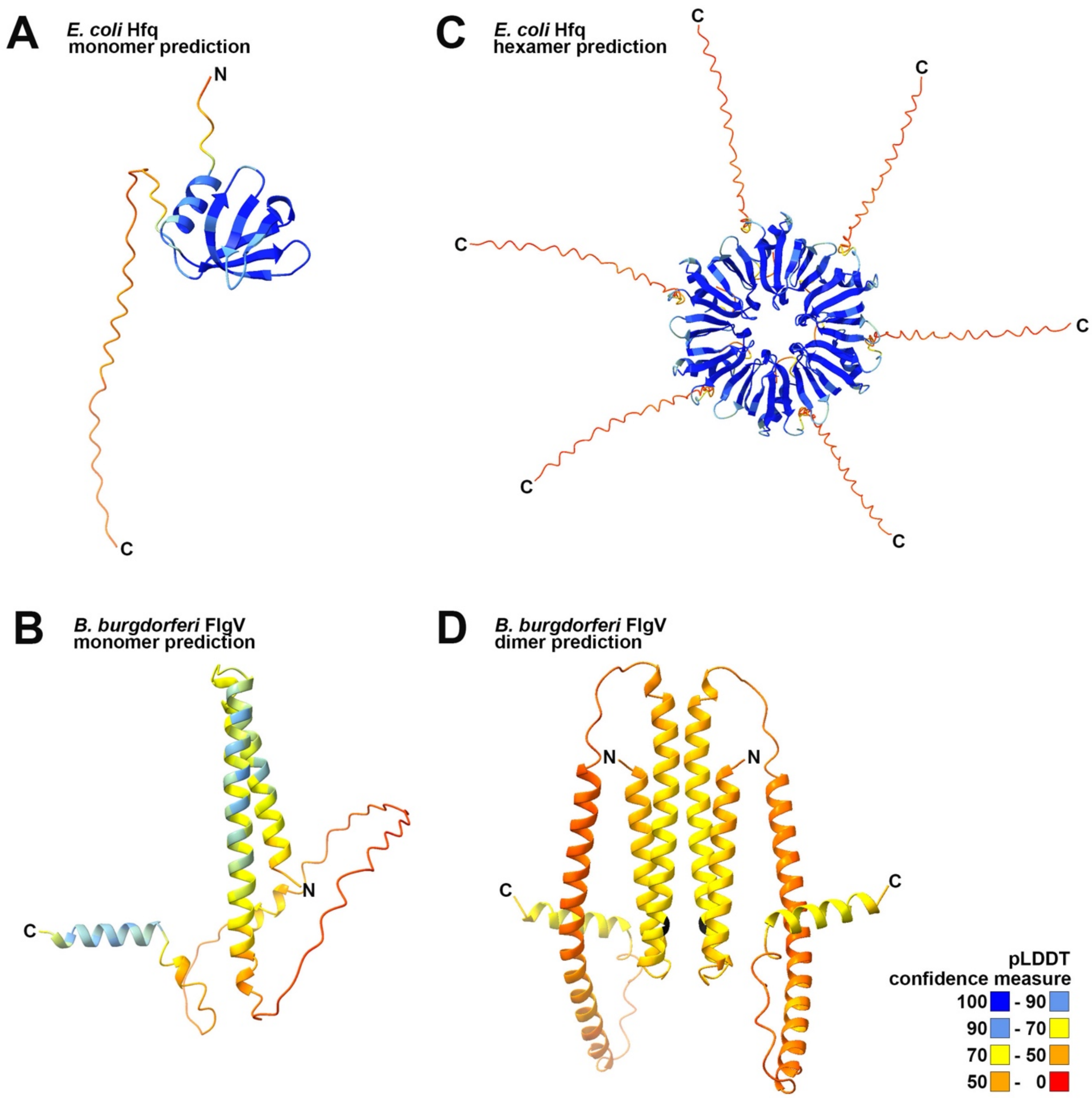

Fig. S3. AlphaFold 3 structure predictions for *E. coli* Hfq and *B. burgdorferi* BB0268 (FlgV). Amino acid sequences analyzed by AlphaFold 3[1] for: (**A**) *E. coli* Hfq monomer; (**B**) *B. burgdorferi* BB0268 (FlgV) monomer; (**C**) *E. coli* Hfq hexamer; and (**D**) *B. burgdorferi* FlgV dimer. Structure figures of the AlphaFold 3 output with the highest confidence were prepared using ChimeraX[2]. The structure predictions are colored by the pLDDT confidence measure in the B-factor field. For panel D, the conserved arginine residue (Fig. S6A) is shaded black.

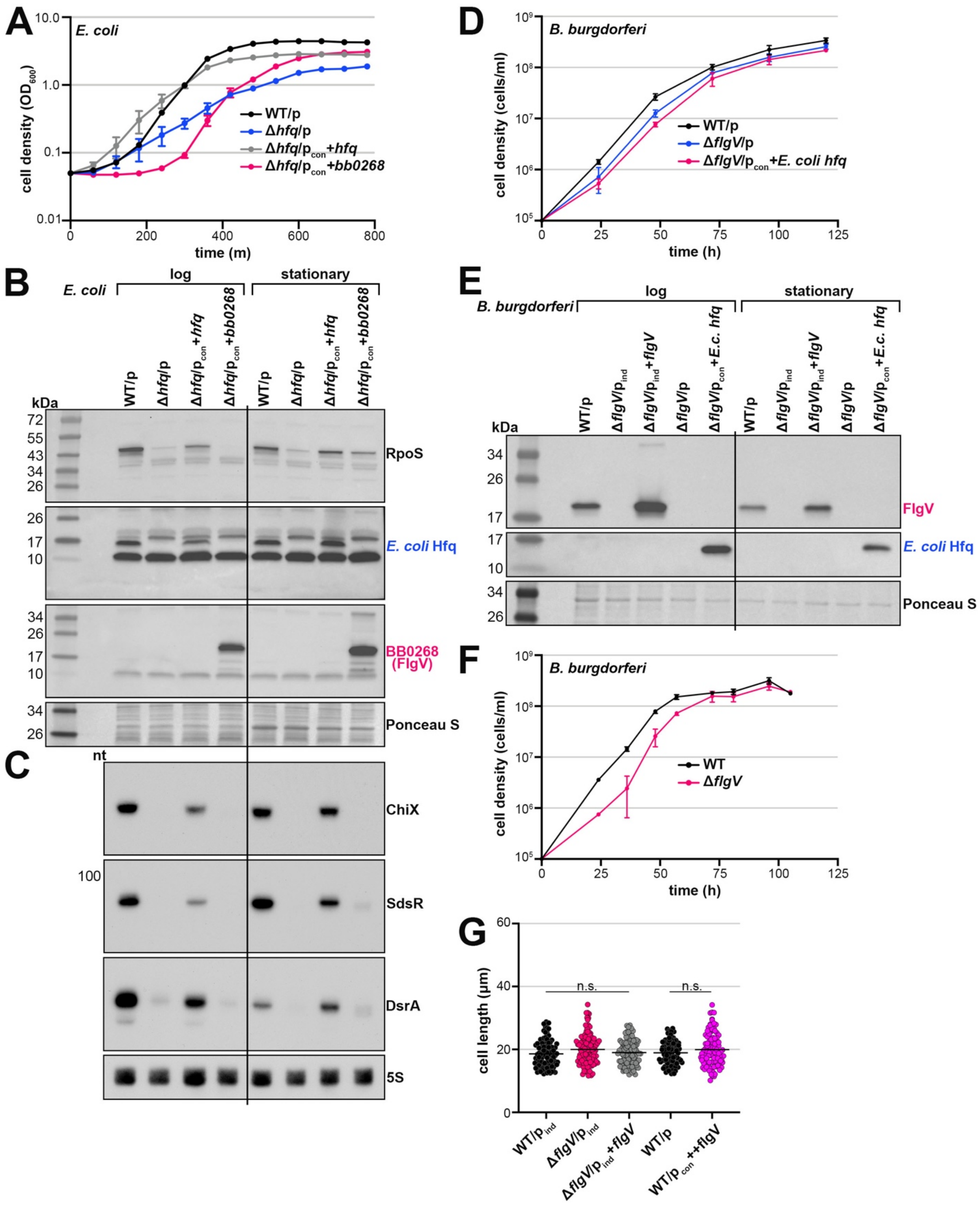

Fig. S4. BB0268 (FlgV) does not complement an *E. coli* Δ*hfq* strain and *E. coli* Hfq does not complement a *B. burgdorferi* Δ*bb0268* (*flgV*) strain. (**A**) Growth curve of Δ*hfq E. coli* expressing *bb0268*. WT (EC013) or Δ*hfq* (EC023) *E. coli* were freshly transformed with the

empty vector, p_{con}+*hfq* and/or p_{con}+*bb0268*. Single colonies were isolated and grown overnight. Cell growth was monitored by OD_{600} every 60 min for 780 min total, after a dilution of the overnight culture to an OD_{600} of 0.05. Each data point (circles) represents the mean of three biological replicates with the standard deviation. (**B**) *E. coli* cultures, defined in panel A, were grown and harvested at log phase (300 min for WT/p and Δ*hfq*/p_{con}+*hfq*; 360 min for Δ*hfq*/p and Δ*hfq*/p_{con}+*bb0268*) and stationary phase (600 min for WT/p and Δ*hfq*/p_{con}+*hfq*; and 720 min for Δ*hfq*/p and Δ*hfq*/p_{con}+*bb0268*). Protein extracts were separated on a Tris-Glycine gel, transferred to a membrane, stained with Ponceau S as a loading control, and probed using α-RpoS, α-*E.coli*-Hfq, and α-BB0268(FlgV) antibodies. Proteins were probed sequentially on the same membrane; size markers are indicated. (**C**) Northern analysis of the *E. coli* ChiX, SdsR and DsrA sRNAs. RNA extracts isolated from the same cultures in panel B were separated on an acrylamide gel, transferred to a membrane and probed for specific sRNAs (RNAs were probed sequentially on the same membrane) and 5S as a loading control. Size markers are indicated for all RNAs, except where the portion of the blot shown is less than 100 nt. (**D**) Growth curve of Δ*flgV*(*bb0268*) *B. burgdorferi* expressing *E. coli* Hfq. Cell growth was monitored by dark field microscopy enumeration at the indicated time points, after dilution of the starter cultures (PA023, PA257, PA261) to $1x10^5$ cells/ml. Each data point (circles) represents the mean of three biological replicates with the standard deviation. (**E**) Immunoblot analysis of BB0268 and *E. coli* Hfq levels in *B. burgdorferi*. WT/p_{ind} (PA273), Δ*flgV*/p_{ind} (PA310), and Δ*flgV*/p_{ind}+*flgV* (PA312) were grown with 0.1 mM IPTG, after dilution of the starter culture, to an average density of ~$1.1x10^7$ cells/ml (log) and ~$2.3x10^8$ cells/ml (stationary), and total protein was isolated. Δ*flgV*/p (PA257) and Δ*flgV*/p_{con}+*E.coli*(*E.c.*) *hfq* (PA261) were grown, after dilution of the starter culture, to an average density of ~$1.4x10^7$ cells/ml (log) and ~$2.8x10^8$ cells/ml (stationary), and total protein was isolated. Δ*flgV*/p_{ind} (PA310), Δ*flgV*/p (PA257) and Δ*flgV*/p_{con}+*E.coli*(*E.c.*) *hfq* (PA261) samples were collected 9 h after the WT/p_{ind} (PA273) and Δ*flgV*/p_{ind}+*flgV* (PA312) samples, so that all cultures were collected at the same cell density.

Protein extracts were separated on a Tris-Glycine gel, stained with Ponceau S as a loading control, and probed with α-FlgV and α-*E.coli*-Hfq antibodies. A subset of the lanes is shown in Fig. 3A. Proteins were probed sequentially on the same membrane; size markers are indicated. (**F**) Growth curve of WT and Δ*flgV*(*bb0268*) *B. burgdorferi*. Cell growth was monitored by dark field microscopy enumeration at the indicated time points, after dilution of the starter cultures (PA001 and PA011) to $1x10^5$ cells/ml. Each data point (circles) represents the mean of three biological replicates with the standard deviation. Lag phase growth rate (~$1x10^5$ to ~$3x10^6$ cells/ml) and double time was calculated for each biological replicate and averaged: WT = 4.6 h; Δ*flgV* 8.5 h. Average growth rates were significantly different, compared by a student t-test, GraphPad Prism 9.5.1 (p = 0.0159). (**G**) Quantification of spirochete length, for the strains in Fig. 3D–I, at stationary phase. All cultures were grown to an average density of ~$1.8x10^8$ cells/ml, washed with 1X PBS and imaged. Approximately 100 cells were traced using the curve (spline) tool with ZEN 3.4 (blue edition) software. Each data point (circles) represents the length of one spirochete; the line indicates the mean length for each strain. Average length across WT/p_{ind}, Δ*flgV*/p_{ind}, and Δ*flgV*/p_{ind}+*flgV* samples or WT/p and WT/p_{con}++*flgV* were compared by one-way ANOVA with Tukey's multiple comparisons test or t test, respectively, GraphPad Prism 9.5.1 (n.s., not significant).

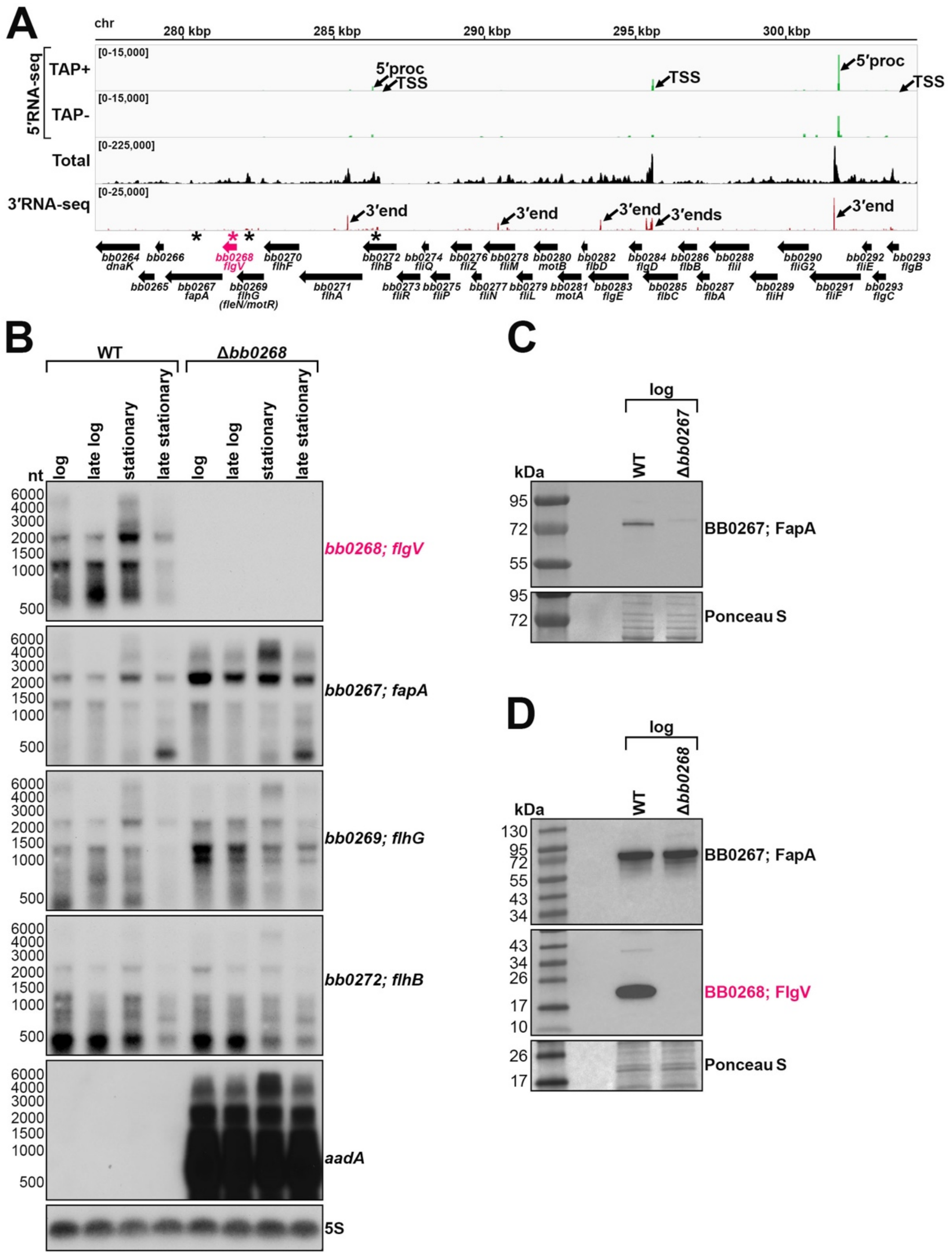

Fig. S5. ***bb0268* (*flgV*) is co-transcribed with flagellar genes.** (**A**) RNA-seq browser image of the "*flgB* superoperon", including the *bb0268* (*flgV*) locus. Browser images display sequencing reads for one DNA strand for RNA isolated from logarithmic phase cells. 5′RNA-seq tracks (green) represent an overlay of two biological replicates[3], while total (black) and 3′RNA-seq

(red) tracks represent an overlay of three biological replicates[4]. Read count ranges are shown in the top left of each frame. The chromosomal coordinates and relative orientation of ORFs (wide black arrows) are depicted. Select transcription start sites (TSS; as determined by the ratio of reads between ±TAP tracks), 5′ processed ends (5′proc) and 3′ ends are indicated (small black arrows). (**B**) Northern analysis of *bb0268* (*flgV*) across growth. Strains PA001 and PA011 were harvested at time points after the dilution of the subculture for spirochetes in logarithmic phase (log): WT $1.6x10^7$ cells/ml, 36 h; Δ*bb0268* $1.5x10^7$ cells/ml, 48 h; late logarithmic phase (late log): WT $7.5x10^7$ cells/ml, 48 h; Δ*bb0268* $7.8x10^7$ cells/ml, 57 h; stationary phase (stationary): WT $1.7x10^8$ cells/ml, 72 h; Δ*bb0268* $1.9x10^8$ cells/ml, 81 h; late stationary phase (late stationary): WT $3.7x10^8$ cells/ml, 96 h; Δ*bb0268* $1.7x10^8$ cells/ml, 105 h. For each timepoint, total RNA was isolated, separated on an agarose gel, transferred to a membrane and probed for the indicated RNAs. Approximate probe sequence location is indicated by the pink (*bb0268*) or black (*bb0267*; *bb0269*, *bb0272*) asterisks on the corresponding browser image (panel A); size markers are indicated. RNAs were probed sequentially on the same membrane. The membrane was also probed for 5S as a loading control. (**C**) Immunoblot analysis of FapA levels in WT (PA001) and *fapA* (*bb0267*) (PA395) deletion. Protein extracts were separated on a Tris-Glycine gel, transferred to a membrane, stained with Ponceau S as a loading control, and probed with α-FapA antibodies. Size markers are indicated. (**D**) Immunoblot analysis of FapA and FlgV levels in WT and *flgV* deletion. WT (PA001) and Δ*flgV* (PA011) were grown to an average density of ~$1.7x10^7$ cells/ml (log) after dilution of the starter culture. Immunoblot analysis was performed as in panel C.

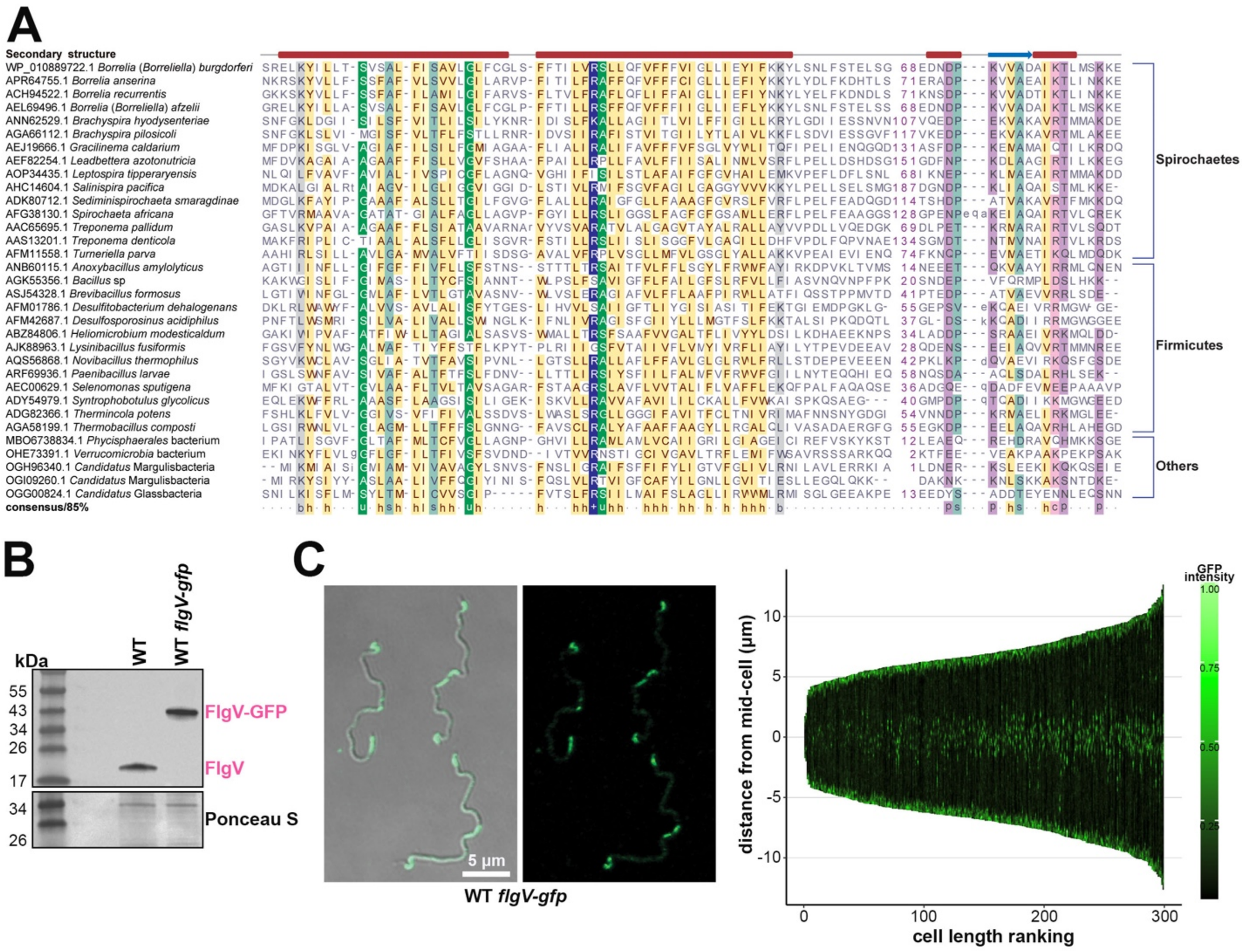

Fig. S6. FlgV is a conserved, membrane-associated protein. (A) A multiple sequence alignment of the newly identified Type-1 FlgV clade from spirochetes, firmicutes (Bacillota) and other lineages. The sequences are labeled using their GenBank accession and species name and colored as per the 85% consensus using the following amino acid side chain classes in the following order of priority: +: positive, c: charged, u: tiny, l: aliphatic, h: hydrophobic, p: polar, s: small, b: big. The predicted secondary structure is shown above the alignment. **(B)** Immunoblot analysis of *B. burgdorferi* expressing *gfp*. Total protein was isolated from WT (PA001) and *flgV-gfp* (PA402) *B. burgdorferi*, and protein extracts were separated on a Tris-Glycine gel, stained with Ponceau S as a loading control, and probed with α-FlgV antibodies. Size markers are indicated. (**C**) Localization of FlgV-GFP by confocal microscopy. The same culture of *B. burgdorferi* (PA402) in Fig. 4B was grown to a density of ~$2.6x10^8$ cells/ml, prior to imaging. Left

panels: Bright field and fluorescence composite (left micrograph) and fluorescence-only (right micrograph) are shown. Scale bar on left micrograph also applies to right micrograph. Right panel: Demograph of the GFP profile for 300 spirochetes, cells were positioned in order of increasing length.

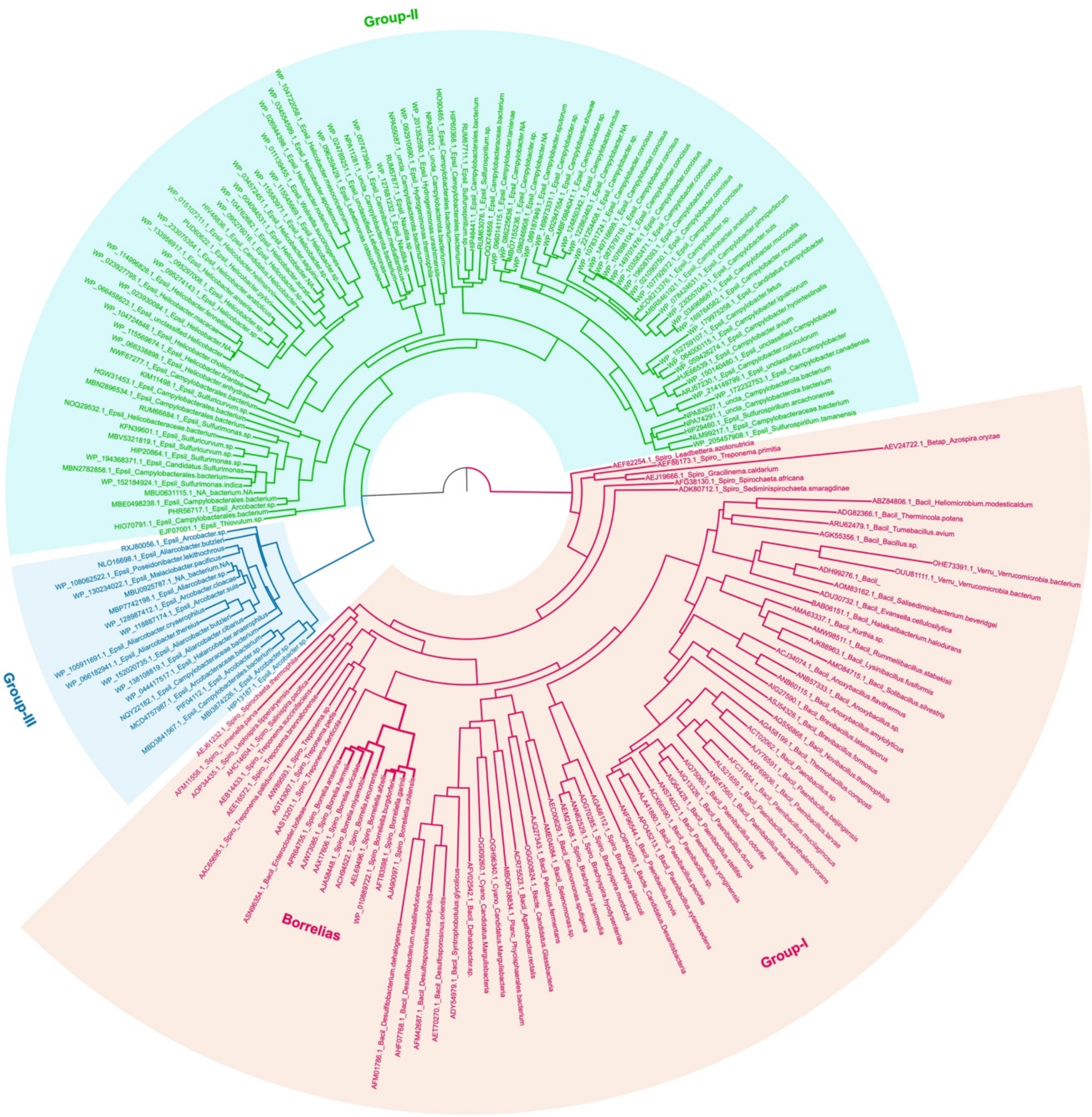

Fig. S7. Phylogenetic analysis of FlgV proteins. A phylogenetic tree of the FlgV trees constructed using the maximum-likelihood method with the LG substitution model with the FastTree program and an input alignment of all the sequences in the tree (c.f. Fig. S6A). The tree was tested for local support of the branches with the Shimodaira-Hasegawa test for branch triplets implemented in FastTree. The three major groups in the above tree were all supported at 100%. Group-I is dominated by Bacillota and Spirochaeta, group-II by classical Campylobacterota (Helicobacteria and Campylobacteria) and group-III by Arcobacter-like Campylobacterota.

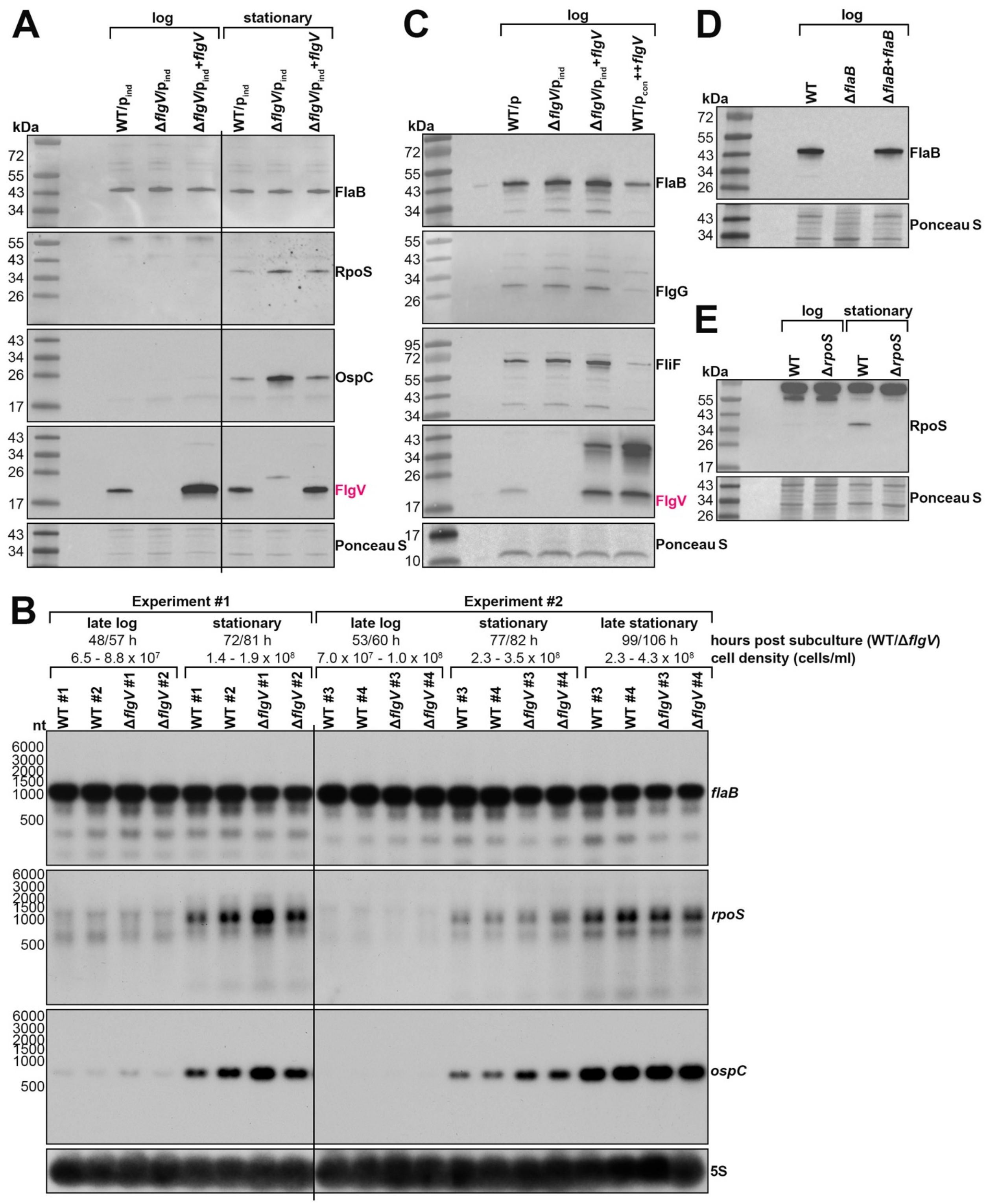

Fig. S8. FlgV levels impact FlaB protein, but not *flaB* mRNA levels. (**A**) Immunoblot analysis of FlaB and RpoS when FlgV is deleted. Protein extracts from the same samples in Fig. 3A were

separated on an independent Tris-Glycine gel, stained with Ponceau S as a loading control, and probed with α-FlaB[5], α-RpoS (this study; see panel E), α-OspC and α-FlgV antibodies. Proteins were probed sequentially on the same membrane; size markers are indicated. **(B)** Northern analysis of the *flaB*, *rpoS*, and *ospC* mRNAs. RNA extracts isolated from two independent experiments, each with two biological replicates of WT (PA001) and Δ*bb0268* (PA011) cultures, were separated on an agarose gel, transferred to a membrane and probed for specific mRNAs (RNAs were probed sequentially on the same membrane) and 5S as a loading control. The cell density and time of cell collection (WT/Δ*flgV*), after a subculture to 1×10^5 cells/ml, is indicated above the samples. Size markers are indicated. (**C**) Immunoblot analysis of flagellar structural proteins when *flgV* is deleted or the protein is overproduced. Cells were grown to a density of: 3.75×10^7 cells/ml for WT/p (PA023), 2.25×10^7 cells/ml for Δ*flgV*/p_{ind} (PA310), 4.25×10^7 cells/ml for Δ*flgV*/p_{ind}+*flgV* (PA312), and 0.90×10^7 cells/ml for WT/p_{ind}++*flgV* (PA267) *B. burgdorferi*. Cells were lysed by sonication and protein extracts were separated on a Tris-Glycine gel, stained with Ponceau S as a loading control, and probed with α-FlaB (this study; see panel D), α-FlgG, α-FliF, and α-FlgV antibodies. Proteins were probed sequentially on the same membrane; size markers are indicated. (**D**) Specificity of the FlaB antibody. Cells were grown to a density of: 4.00×10^7 cells/ml for WT (PA001), 3.00×10^7 cells/ml for Δ*flaB* (PA365;[6]), and 1.65×10^7 cells/ml for Δ*flaB*+*flgV* (PA367;[7]) *B. burgdorferi*. Protein extracts were separated as described in panel A and probed with α-FlaB (this study). (**E**) Specificity of the RpoS antibody. Cells were grown to a density of: 4.50×10^7 cells/ml for WT (PA001) and 3.50×10^7 cells/ml for Δ*rpoS* (PA235) *B. burgdorferi* for the logarithmic phase samples and 3.0×10^8 cells/ml for WT (PA001) and 2.65×10^8 cells/ml for Δ*rpoS* (PA235) *B. burgdorferi* for the stationary phase (48 h later) samples. Protein extracts were separated as described in panel A and probed with α-RpoS (this study).

SUPPLEMENTARY REFERENCES

1 Abramson, J. *et al.* Accurate structure prediction of biomolecular interactions with AlphaFold 3. *Nature* (2024). https://doi.org:10.1038/s41586-024-07487-w

2 Meng, E. C. *et al.* UCSF ChimeraX: Tools for structure building and analysis. *Protein Sci* **32**, e4792 (2023). https://doi.org:10.1002/pro.4792

3 Adams, P. P. *et al.* *In vivo* expression technology and 5' end mapping of the *Borrelia burgdorferi* transcriptome identify novel RNAs expressed during mammalian infection. *Nucleic Acids Res* **45**, 775-792 (2017). https://doi.org:10.1093/nar/gkw1180

4 Petroni, E. *et al.* Extensive diversity in RNA termination and regulation revealed by transcriptome mapping for the Lyme pathogen *Borrelia burgdorferi*. *Nat Commun* **14**, 3931 (2023). https://doi.org:10.1038/s41467-023-39576-1

5 Barbour, A. G., Hayes, S. F., Heiland, R. A., Schrumpf, M. E. & Tessier, S. L. A *Borrelia*-specific monoclonal antibody binds to a flagellar epitope. *Infect Immun* **52**, 549-554 (1986).

6 Motaleb, M. A. *et al.* *Borrelia burgdorferi* periplasmic flagella have both skeletal and motility functions. *Proc Natl Acad Sci U S A* **97**, 10899-10904 (2000). https://doi.org:10.1073/pnas.200221797

7 Sartakova, M. L. *et al.* Complementation of a nonmotile *flaB* mutant of *Borrelia burgdorferi* by chromosomal integration of a plasmid containing a wild-type *flaB* allele. *J Bacteriol* **183**, 6558-6564 (2001). https://doi.org:10.1128/JB.183.22.6558-6564.2001
