# Supplementary material for: Broadly conserved FlgV controls flagellar assembly and Borrelia burgdorferi dissemination in mice
Source: Nat Commun. 2024 Nov 29;15:10417. doi: 10.1038/s41467-024-54806-w (PMC11607428; doi:10.1038/s41467-024-54806-w)
Supplement: Supplementary file 2 — Description of Additional Supplementary Files [file 41467_2024_54806_MOESM2_ESM.pdf]

Description of Additional Supplementary Files:

Supplementary Data 1: List of strains, plasmids, oligonucleotides, and synthesized genes used in this study

Supplementary Movie 1: Three-dimensional reconstruction from a WT/p (PA023) *B. burgdorferi*.

Supplementary Movie 2: Three-dimensional reconstruction from a Δ*flgV*/p_{ind} (PA310) *B. burgdorferi*.

Supplementary Movie 3: Three-dimensional reconstruction from a Δ*flgV*/p_{ind}+*flgV* (PA312) *B. burgdorferi*.

Supplementary Movie 4: Three-dimensional reconstruction from a WT/p_{con}++*flgV* (PA267) *B. burgdorferi*.
