# Supplementary material for: Broadly conserved FlgV controls flagellar assembly and Borrelia burgdorferi dissemination in mice
Source: Nat Commun. 2024 Nov 29;15:10417. doi: 10.1038/s41467-024-54806-w (PMC11607428; doi:10.1038/s41467-024-54806-w)
Supplement: Supplementary file 8 — Reporting Summary [file 41467_2024_54806_MOESM8_ESM.pdf]

nature portfolio

Corresponding author(s): Philip P. Adams

Last updated by author(s): Oct 15, 2024

Reporting Summary

Nature Portfolio wishes to improve the reproducibility of the work that we publish. This form provides structure for consistency and transparency in reporting. For further information on Nature Portfolio policies, see our Editorial Policies and the Editorial Policy Checklist.

Statistics

For all statistical analyses, confirm that the following items are present in the figure legend, table legend, main text, or Methods section.

n/a	Confirmed	
☐	☒	The exact sample size (*n*) for each experimental group/condition, given as a discrete number and unit of measurement
☐	☒	A statement on whether measurements were taken from distinct samples or whether the same sample was measured repeatedly
☐	☒	The statistical test(s) used AND whether they are one- or two-sided<br>*Only common tests should be described solely by name; describe more complex techniques in the Methods section.*
☒	☐	A description of all covariates tested
☒	☐	A description of any assumptions or corrections, such as tests of normality and adjustment for multiple comparisons
☐	☒	A full description of the statistical parameters including central tendency (e.g. means) or other basic estimates (e.g. regression coefficient) AND variation (e.g. standard deviation) or associated estimates of uncertainty (e.g. confidence intervals)
☐	☒	For null hypothesis testing, the test statistic (e.g. *F*, *t*, *r*) with confidence intervals, effect sizes, degrees of freedom and *P* value noted<br>*Give P values as exact values whenever suitable.*
☒	☐	For Bayesian analysis, information on the choice of priors and Markov chain Monte Carlo settings
☒	☐	For hierarchical and complex designs, identification of the appropriate level for tests and full reporting of outcomes
☒	☐	Estimates of effect sizes (e.g. Cohen's *d*, Pearson's *r*), indicating how they were calculated

Our web collection on statistics for biologists contains articles on many of the points above.

Software and code

Policy information about availability of computer code

Data collection	Protein sequence-profile searches were performed with the PSI-BLAST program against the NCBI non-redundant (nr) database clustered down to 50% sequence identity using the MMSEQS program with a profile-inclusion threshold was set at an e-value of 0.01. Iterative HMM searches were performed using the JACKHMMER program from the HMMER3 package.
Data analysis	Protein sequence alignments were performed using the FAMSA and MAFFT programs. TM segments and signal peptides were predicted using a deep neural network as implemented in SignalP 6 and HMM-based TMHMM and Phobius programs. Protein secondary structures were inferred using the JPred3 program with MSAs as inputs. Structural models were generated using the RoseTTAfold or AlphaFold3.

For manuscripts utilizing custom algorithms or software that are central to the research but not yet described in published literature, software must be made available to editors and reviewers. We strongly encourage code deposition in a community repository (e.g. GitHub). See the Nature Portfolio guidelines for submitting code & software for further information.

Data

Policy information about availability of data

All manuscripts must include a data availability statement. This statement should provide the following information, where applicable:

- Accession codes, unique identifiers, or web links for publicly available datasets
- A description of any restrictions on data availability
- For clinical datasets or third party data, please ensure that the statement adheres to our policy

Source data for this paper have been submitted to Figshare (https://doi.org/10.6084/m9.figshare.27005029), which includes all uncropped and unprocessed scans

with molecular weight markers labeled.

Research involving human participants, their data, or biological material

Policy information about studies with human participants or human data. See also policy information about sex, gender (identity/presentation), and sexual orientation and race, ethnicity and racism.

Reporting on sex and gender	not applicable
Reporting on race, ethnicity, or other socially relevant groupings	not applicable
Population characteristics	not applicable
Recruitment	not applicable
Ethics oversight	not applicable

Note that full information on the approval of the study protocol must also be provided in the manuscript.

Field-specific reporting

Please select the one below that is the best fit for your research. If you are not sure, read the appropriate sections before making your selection.

[x] Life sciences   [ ] Behavioural & social sciences   [ ] Ecological, evolutionary & environmental sciences

For a reference copy of the document with all sections, see nature.com/documents/nr-reporting-summary-flat.pdf

Life sciences study design

All studies must disclose on these points even when the disclosure is negative.

Sample size	All experiments were performed in biological duplicate or triplicate.
Data exclusions	No data were excluded.
Replication	Number of repeats for each experiment are reported in the figure legends, when appropriate. We observed the same results across independent experiments (performed at least twice).
Randomization	No randomization of samples was used in our study.
Blinding	Investigators were not blinded. The investigators had no bias towards the outcome of the life study experiments and everything was performed openly with eager anticipation for any results obtained from the life study experiments.

Reporting for specific materials, systems and methods

We require information from authors about some types of materials, experimental systems and methods used in many studies. Here, indicate whether each material, system or method listed is relevant to your study. If you are not sure if a list item applies to your research, read the appropriate section before selecting a response.

Materials & experimental systems

n/a	Involved in the study	
[ ]	[x]	Antibodies
[x]	[ ]	Eukaryotic cell lines
[x]	[ ]	Palaeontology and archaeology
[ ]	[x]	Animals and other organisms
[ ]	[ ]	Clinical data
[x]	[ ]	Dual use research of concern
[x]	[ ]	Plants

Methods

n/a	Involved in the study	
[x]	[ ]	ChIP-seq
[x]	[ ]	Flow cytometry
[x]	[ ]	MRI-based neuroimaging

Antibodies

Antibodies used	Generated in this study: polyclonal antibodies to FlgV, FapA, FlaB, and RpoS were generated by immunizing rabbits with purified His-tagged recombinant FlgV protein (amino acids 58–159), recombinant FapA, recombinant FlaB, or recombinant RpoS protein followed by affinity purification (GenScript).

	Acquired antibodies: α-FLAG-HRP (Sigma-Aldrich), α-RpoS-E.coli (gift from Sue Wickner, NIH), α-Hfq (PMID:11804582), α-FlaB (PMID:3516878), α-FlgG (PMID: 32039533 & 23940315), α-FliF (PMID: 32039533), α-OspC (Rockland; Cat. #200-401-C11), α-SodA (PMID: 19040638), peroxidase labeled α-mouse (GE Healthcare; Cat. #NIF825), peroxidase labeled α-rabbit (GE Healthcare; Cat. #NIF824), and peroxidase labeled α-rat (Invitrogen; Cat. #62-9520).
Validation	Immunoblot analyses using B. burgdorferi lysates and probing for FlgV (Fig. 3A), FapA (Fig. S5C), FlaB (Fig. S8D) and RpoS (Fig. S8E) confirmed the specificity of the antibodies generated in this study.

Animals and other research organisms

Policy information about studies involving animals; ARRIVE guidelines recommended for reporting animal research, and Sex and Gender in Research

Laboratory animals	Mice: C3H/HeN (Envigo). 6-8 week old mice were used for all experiments. Mice were housed at 72°F; 50-70% humidity with a 12/12hour light/dark cycle.<br><br>Ticks: Ixodes scapularis (Oklahoma State University). Larval and Nymphal ticks were used for all experiments. Ticks were stored in a 23°C incubator under 98% humidity.
Wild animals	Study did not involve wild animals.
Reporting on sex	Female mice were used for the B. burgdorferi infection.
Field-collected samples	Study did not involve field-collected samples.
Ethics oversight	The National Institutes of Health and the University of Central Florida are accredited by the International Association for Assessment and Accreditation of Laboratory Animal Care. All animals were cared for in compliance with the Guide for the Care and Use of Laboratory Animals. Protocols for all animal experiments were prepared according to the guidelines of the National Institutes of Health, reviewed and approved by the Eunice Kennedy Shriver National Institute of Child Health and Human Development Institutional Animal Care and Use Committee or the UCF Institutional Animal Care and Use Committee.

Note that full information on the approval of the study protocol must also be provided in the manuscript.

Clinical data

Policy information about clinical studies
All manuscripts should comply with the ICMJE guidelines for publication of clinical research and a completed CONSORT checklist must be included with all submissions.

Clinical trial registration	*Provide the trial registration number from ClinicalTrials.gov or an equivalent agency.*
Study protocol	*Note where the full trial protocol can be accessed OR if not available, explain why.*
Data collection	*Describe the settings and locales of data collection, noting the time periods of recruitment and data collection.*
Outcomes	*Describe how you pre-defined primary and secondary outcome measures and how you assessed these measures.*

Plants

Seed stocks	*Report on the source of all seed stocks or other plant material used. If applicable, state the seed stock centre and catalogue number. If plant specimens were collected from the field, describe the collection location, date and sampling procedures.*
Novel plant genotypes	*Describe the methods by which all novel plant genotypes were produced. This includes those generated by transgenic approaches, gene editing, chemical/radiation-based mutagenesis and hybridization. For transgenic lines, describe the transformation method, the number of independent lines analyzed and the generation upon which experiments were performed. For gene-edited lines, describe the editor used, the endogenous sequence targeted for editing, the targeting guide RNA sequence (if applicable) and how the editor was applied.*
Authentication	*Describe any authentication procedures for each seed stock used or novel genotype generated. Describe any experiments used to assess the effect of a mutation and, where applicable, how potential secondary effects (e.g. second site T-DNA insertions, mosiacism, off-target gene editing) were examined.*
